# A Review of 3D Printed Bone Implants

**DOI:** 10.3390/mi13040528

**Published:** 2022-03-27

**Authors:** Zhaolong Li, Qinghai Wang, Guangdong Liu

**Affiliations:** 1 Key Laboratory of Advanced Manufacturing Intelligent Technology of Ministry of Education, Harbin University of Science and Technology, Harbin 150080, China; lizhaolong@hrbust.edu.cn (Z.L.); wangqinghai3715@163.com (Q.W.); 2 Harbin Vocational & Technical College, Harbin 150001, China

**Keywords:** 3D printing, bioprinting, bone implant, biomaterials, bone forming technology

## Abstract

3D printing, that is, additive manufacturing, has solved many major problems in general manufacturing, such as three-dimensional tissue structure, microenvironment control difficulty, product production efficiency and repeatability, etc., improved the manufacturing speed and precision of personalized bone implants, and provided a lot of support for curing patients with bone injuries. The application of 3D printing technology in the medical field is gradually extensive, especially in orthopedics. The purpose of this review is to provide a report on the related achievements of bone implants based on 3D printing technology in recent years, including materials, molding methods, optimization of implant structure and performance, etc., in order to point out the existing shortcomings of 3D printing bone implants, promote the development of all aspects of bone implants, and make a prospect of 4D printing, hoping to provide some reference for the subsequent research of 3D printing bone implants.

## 1. Introduction

Human bones have the ability to self-repair and regenerate, but this ability is limited [1,2]. When the bone damage exceeds its acceptable capacity, it will lose its self-healing function and require artificial repair. The ideal treatment is to repair the damaged area by transplanting bones from other parts of the patient himself, thus possessing the same bone conduction ability. The application of this method is limited by the limitation of transplantable bones at the donor site of patients and the possible complications at the donor site after transplantation [3]. The second reason is that the donor bone is from other people, so that enough donors can be obtained. However, there is often non-specific immunity to reject the external donor, which poses great difficulties for bone repair [4,5]. Therefore, in order to overcome these limitations of traditional bone repair, researchers started to study the possibility of replacing human bones with grafts. 3D printing technology plays a huge role in the biomedical field due to its unique advantages. Researchers use cells and biomedical materials as discrete materials and make use of the characteristics of personalized customization of 3D printing to prepare different organs and tissue structures, which will solve the problem of insufficient organ donors to a large extent. In particular, for applications in bones and bone scaffolds, 3D printing offers a solution for the treatment of patients with complex bone defects. The intersection of 3D printing technology and the biomedical field will surely become a highlight of modern medicine. In the application process of 3D printing, in addition to the limitation of its own technology, the choice of printing materials also plays a vital role. Currently, the materials used for 3D printing of biological bones and bone scaffolds include metal materials (such as titanium alloy and magnesium alloy), inorganic non-metal materials (such as biological glass, biological ceramic and biological cement), and high-molecular materials (such as polycaprolactone, polylactic acid and poly-ether-ether-ketone) [6,7]. Human bone is a composite material, so bone implants are mostly made of composite materials, which can simulate the structure and performance of natural bone as much as possible. The 3D printing technologies mainly include extrusion, inkjet, laser melting and laser sintering. Extrusion-based printing is currently the most popular. This technology allows multiple materials to bind to cells individually or in the form of biological ink [8,9,10]. In order to make the mechanical properties and elastic modulus of the bone graft closer to that of natural bone and avoid the stress rejection or insufficient conduction ability after transplantation, researchers optimized the internal structure of the bone graft and modified the materials used and the printed surface of bone tissue. This review summarizes the current status of research on the optimization of materials, molding methods, structure and properties of bone implants, and prospects the development trend of 3D printing technology in the field of orthopedics, aiming to provide a reference for future research in related fields.

## 2. Materials

Natural bone is a highly organized composite material, including about 60% inorganic components. Its main components are hydroxyapatite crystals and about 30% organic materials, mainly composed of type I collagen and a small amount of type V collagen, 5–10% water and 3% lipid. Natural human bones are structurally divided into periosteum, compact bone, cancellous bone and bone marrow [11]. Osteoblasts form new bone tissue in the form of osteoids. Osteoids are composed of collagen and other proteins [12]. Biocompatibility of bone implant materials and their degradation products is an important factor emphatically considered in bone tissue engineering. Bone implants should allow attachment, proliferation, and differentiation of cells without negatively affecting the cells and minimizing the immune response associated with non-identical transplants. For BTE (bone tissue engineering), bioactivity covers two primary biological processes: osteo-conductivity and osteo-inductivity [13]. At present, many materials have been used in BTE; their characteristics are shown in Table 1.

### 2.1. Inorganic Biomaterials

Inorganic biomaterials, such as metals and bio-ceramics, have been widely used to repair and regenerate diseased and damaged bones. This type of biomaterial is particularly useful for bone transplantation and bone cement, orthopedic load-bearing coatings (acetabular cups), and periodontal repair.

#### 2.1.1. Non-Degradable Metal Material

Titanium and its alloys are mild, have a low elastic modulus, low density, good corrosion resistance, and the dense passivation film on its surface gives it good biocompatibility, which is currently the most widely used medical metal [27]. At present, pure titanium and TC4 titanium alloy (Ti6A14V) have been comparatively mature in research. Researchers are developing titanium alloys with better biocompatibility and a lower elastic modulus, such as Ti-6A1-7Nb, Ti-13Nb-13Zr and Ti-35Nb-5Ta-7Zr [28].

The elastic modulus of pure titanium is 110 GPa, which is lower than that of stainless steel and other metals, but far higher than that of bone. Therefore, the titanium alloy implant prepared by L-PBF has a porous structure. By adjusting the porosity, its elastic modulus can be close to that of bone, thus avoiding the stress shielding effect. In addition to the forming quality and microstructure, the pore design of the porous structure will also significantly affect the mechanical properties [29,30]. You et al. [31] printed a titanium multi-tooth implant with a porous structure, with pores of 300–400 μm. Through animal experiments and comparison, bone tissue grows into the pores on the surface of the implant, and has high bone tissue density, which indicates that the 3D printed tooth implant has good osseointegration ability. Alberto et al. [32] used Ti6Al4V to prepare porous bone scaffold by EBM technology. Experiments show that porous scaffold has good biocompatibility and mechanical properties and can promote bone growth and bone fusion. The author proposed a new reconstruction technique, that is, customized bionic porous titanium scaffold by electron beam melting to treat large bone defects. Experiments have proved the potential of porous Ti6Al4V scaffold made of additive as a bone defect repair device. Shi et al. [33] used Ti6Al4V powder as the raw material to print out porous titanium artificial bone in 3D, and its structure showed uniform honeycomb structure, with a micropore size less than 10 μm, porosity of 70.56%, compressive strength of 194 MPa and bending strength of 105 MPa, and its structure and performance could meet the use requirements of human bones. Zhang [34] successfully prepared a 3D printed Ti6Al4V4Cu alloy material. Through the experiment of rabbit femur and ankle bone defect, it was found that compared with Ti6Al4V alloy material, it has better biocompatibility and better osteogenesis performance. Xu et al. [35] prepared a new porous Ti35Zr28Nb scaffold. The scaffold with a porosity of 61.1% showed good mechanical properties, with a compressive yield strength of 132.5 ± 3.5 MPa and elastic modulus of 2.9 ± 0.4 GPa, which was very suitable for replacing natural bones. In vitro cell experiments show that it is non-toxic. Wang et al. [36] made a porous functionally gradient scaffold (PFGS) with Ti6Al4V as the raw material by SLM, and it was found that its mechanical properties were close to those of natural bones. The cell activity test showed that the cell proliferation rate was 140% on the 4th to 7th day, which was suitable for bone tissue implantation.

Tantalum has excellent biological inertia and biocompatibility, known as the “biophilic metal”. Since pure tantalum was first applied to orthopedics in 1940, it has exhibited good clinical osteogenic activity, osteo induction and osteo conductivity [37,38]. Wauthle et al. [39] prepared the porous tantalum implant via SLM, which has high porosity, a high elastic modulus, and no cytotoxicity, thus meeting the requirements for bone implant materials. Animal experiments have shown that significant bone in-growth occurred aft that implant was implanted into the body, which formed a good combination with the bone interface, proving that the 3D printed porous tantalum implant had excellent mechanical properties, biocompatibility and osteo conductivity, and could be used to repair and treat bone defects. At present, the research on the fabrication of porous support by adding materials is at an initial stage, and the preparation of powder materials and special equipment are the main problems that limit the current large-scale development of personalized porous support implants. However, the fabrication technology of adding materials such as laser powder bed fusion (LPBF) is expected to become an important way to fabricate personalized porous support implants in the future due to its high degree of freedom and efficiency.

#### 2.1.2. Degradable Metal Material

Degradable metals can be gradually degraded by body fluid corrosion in the body, and the released corrosion products can bring about an appropriate host reaction in the body. After assisting the body to complete the mission of tissue repair, they will all be absorbed or excreted by the body [40]. An ideal degradable metal should meet at least three conditions: the degradation products are not harmful to the human body. The rate of degradation is matched to the rate of healing of the organism. It has excellent mechanical properties and can provide enough mechanical support in the early stage. According to the mass fraction of metal elements in the human body, we can know that calcium, potassium, sodium, magnesium, iron and zinc have the most content and the best biocompatibility. However, calcium, potassium and sodium are too active to be prepared into block materials. Therefore, the research on degradable metals is mainly focused on three types of metal materials with iron, magnesium and zinc as the matrix [41,42].

Chou et al. [43] printed the biodegradable bone scaffold using iron-manganese alloy as the raw material, and the scaffold showed similar tensile mechanical properties and good biocompatibility with natural bone. Bose et al. [44] mixed iron oxide and silicon dioxide into β-TCP and formed the scaffold using 3D printing. Animal experiments showed that the addition of Fe^3+^ and Si^4+^ could promote the formation of new bones and blood vessels at an early stage and accelerate the healing of bone defects. Li et al. [45] used magnesium-based slurry as the raw material and prepared the porous titanium bone scaffold using SLS printing technology. It has been determined in many experiments that the porosity of the scaffold is (65.0 ± 2.5) %, the compressive strength is (0.87 ± 0.15) MPa, and the degradation rate of the scaffold is basically stable at (10.0 ± 0.2) mm per year. It is suitable for replacing damaged bones. Lai et al. [46] used magnesium (Mg) powder, PLGA, and β-TCP as the raw materials, and adopted low-temperature 3D printing technology to prepare a novel porous PLGA/TCP/Mg (PTM) scaffold. The steroid-related osteonecrosis experiment of rabbits showed that PTM could significantly promote new bone formation and angiogenesis, exhibited good mechanical properties, and was a promising composite biomaterial. Zhang et al. [47] prepared a composite scaffold containing magnetic ferro ferric oxide nanoparticles and bioactive glass/polycaprolactone so that the porosity of the scaffold was uniformly distributed at 60%, the diameter was 400 μm, and the compressive strength was 13–16 MPa, and loaded anticancer drugs into the scaffold to promote osteoblast osteogenesis, while achieving continuous drug delivery. The experimental results showed that the 3D printed biodegradable porous scaffold made of iron-based alloy could promote bone regeneration without any complications.

**Figure 1:**
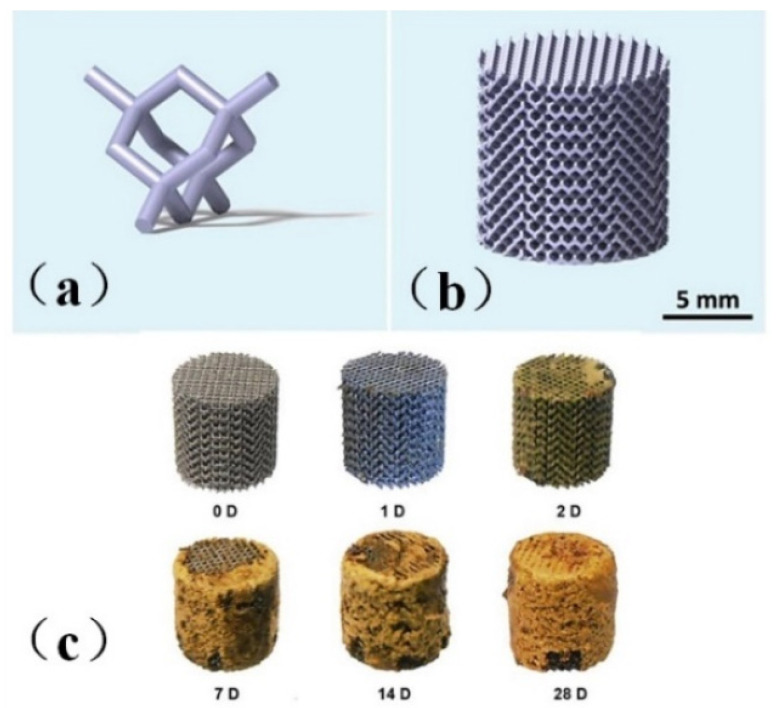
Model drawing of bone scaffold [48]. (**a**) unit cell structure (**b**) bone scaffold structure diagram (**c**) Bone scaffold degradation.

Li et al. [48] printed topologically ordered porous iron scaffolds using metal, as shown in Figure 1a,b and studied the effect of the direct metal printing process on the surface area and grain size of the scaffolds. The results showed that the mechanical properties of the porous scaffolds remained within the allowable range of trabecular bone even after four weeks of degradation, with a degradation rate 12 times faster than that of cold-rolled iron, a mass loss of only 3.1%, and the 28-day degradation process was shown in Figure 1c. The author introduced the first report of topologically ordered porous iron made by direct metal printing, and made a comprehensive study on the time evolution of biodegradable behavior, electrochemical performance, biocompatibility and mechanical properties of implants. It was also proved that these implants had mechanical properties simulating bones, accelerated degradation rate and reasonable cell compatibility.

In 2017, Jauer et al. [49] manufactured the WE43 magnesium alloy porous mandibular implant by LPBF technology. In 2018, Li et al. [50] studied the mechanical properties, in vitro degradation behavior and biocompatibility of WE43 magnesium alloy porous scaffold fabricated by LPBF technology, and found that after one month of soaking in simulation body fluid (SBF), the mechanical properties of WE43 scaffold were still excellent, and it can continue to provide mechanical support. The volume loss of the scaffold after 4 weeks of immersion in SBF was 20%. The in vitro cytotoxicity of the scaffold to MG63 cells was less than 25%. In 2019, Li et al. [51] further studied the fatigue behavior of the degradable porous magnesium alloy fabricated by adding materials and the effect of its biodegradation. The results showed that the biodegradation affected the fatigue performance of AM WE43 magnesium alloy stent, and reduced its fatigue strength from 0.36 σ_y_ to 0.26 σ_y_. Therefore, the biodegradable fatigue performance of the AM porous magnesium alloy can be further improved by optimizing the topology design of the porous structure and determining the laser processing parameters of selective laser melting porous material microstructure.

#### 2.1.3. Bio-Ceramic

Most bio-ceramics are rich in more calcium and phosphorus, and can react with bone naturally and firmly. Due to its unique properties different from other biological materials, bio-ceramic can rapidly generate a series of surface reactions at the implantation site, finally leading to the formation of carbonate-containing apatite layer. The biological glass-ceramic has good biocompatibility, and the material can be implanted into the body without rejection, inflammation, tissue necrosis and other reactions, and can form an osseous combination with bone; it has strong binding strength with bone, good interface binding ability and rapid osteogenesis. Pei et al. [52] used HA as the raw material and prepared an HA scaffold with graded macro-pores through 3D printing and microwave sintering technology. Through in vivo testing, new bone formation was observed on the surface of this scaffold, indicating that the HA material was suitable for the preparation of bone implants. In order to improve the biological performance of hydroxyapatite scaffold in bone tissue engineering, Chen et al. [53] used graphite as a pore-forming agent to generate extra micro/nano pores on the basis of macropores, thus generating graded pores. In order to improve the structural similarity between scaffolds and natural bones, they used micron/nano graphite to manufacture scaffolds with different porosity characteristics. The sintering distribution of graphite-treated scaffolds was optimized to reduce the influence of shrinkage. Experiments show that the scaffold with main micropores and a small number of nano-pores formed inside has high clinical application potential due to the improvement of biological activity. Ren et al. [54] provided a new porous hydroxyapatite (HA) scaffold with a 25–30 µm groove structure (HAG). Experiments in animals show that the scaffold has good biocompatibility. HAG scaffold can increase the adsorption of protein and promote the directional growth and expression of osteogenic genes in vitro. Compared with the conventional HA scaffold, the HAG scaffold can significantly promote the osteogenic differentiation of human placenta-derived mesenchymal stem cells (hPMSC) and the maturation of osteoblasts. Therefore, we conclude that the HAG scaffold with groove structure can induce greater bone formation and improve bone formation, which can be used in clinical treatment. Calabrese et al. [55] provided a collagen/HA scaffold with human adipose stem cells (HADSC). The scaffold consists of type I collagen and magnesium-rich hydroxyphosphate stone. Experiments in mice found that collagen/Mg-HA scaffolds implanted subcutaneously in mice can recruit host cells, and these HADSCs can promote osteogenic differentiation and enhance osteoinductive ability and the formation of vascular components after invading the material. They point in the direction of combining native HADSCs with scaffolds, which is good news for the elderly or other specific patient populations with reduced regenerative capacity.

### 2.2. Synthetic Polymer Materials

#### 2.2.1. Degradable Synthetic Polymer Materials

**Figure 2:**
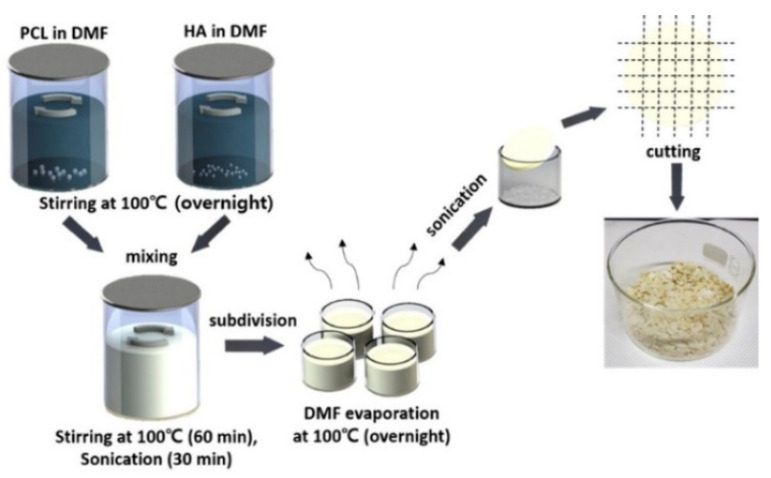
Schematic of the PCL/HA composite flake preparation steps. Photograph showing the PCL/HA composite flakes after evaporating the solvent from the composite solution [64].

Polylactic acid (PLA), polycaprolactone (PCL), polylactic acid-glycolic acid copolymer (PLGA) and other materials are environmentally friendly materials with degradability, and can be obtained by extracting materials from renewable resources in nature through polymerization. These materials have good biocompatibility and mechanical properties. However, a single material always has some defects, such as low surface osteogenic property and high brittleness, which can be overcome after being compounded with some materials to prepare bone implants [56]. Zhang et al. [57] prepared the polylactic acid-hydroxyapatite composite bone marrow stromal cell scaffold, and the results showed that the polylactic acid-hydroxyapatite scaffold had good cell compatibility, and could be used as a bone tissue engineering scaffold for bone repair. Cavo et al. [58] constructed the polylactic acid scaffold using 3D printing technology and applied collagen to the surface of the scaffold, and successfully obtained the relevant theoretical model for the construction of tissue-engineered bone. The microstructure scaffold prepared by Zhang et al. [59] using PCL as the raw material has the function of inducing tissue regeneration and providing a place for cell proliferation, etc. Zhang et al. [60] printed the scaffold using PCL and poly-tri-methylene carbonate (PTMC) as the raw materials, and the results of the manager experiment showed that the composite scaffold had good tissue compatibility. Yu et al. [61] prepared the HA/PCL scaffold, and then co-cultured it with SD rat bone marrow mesenchymal stem cells and found that the composite material had good biocompatibility. Seidenstuecker et al. [62] printed a bone scaffold using BG (bio-glass) and β-TCP powders in different proportions as the raw materials. The experiment showed that the scaffold could support the growth of osteogenic MG-63 cells on the surface of the scaffold material and inside the scaffold material without significant cytotoxicity. The 0/30 bg/β-TCP scaffold exhibited superiority in biocompatibility and mechanical strength among the composites with different proportions. Li et al. [63] tested the influence of dexamethasone on the degradation rate of bone scaffold printed with PLA, PEG (polyethylene glycol) and HA ceramics. It was found that the increase of dexamethasone concentration reduced the degradation rate of scaffold and helped to improve the sustainability of drug release. Kim et al. [64] used PCL and HA to prepare PCL/HA composite sheets for 3D printing bone scaffolds, as shown in the figure. The process of synthesizing PCL/HA composite flakes is displayed in Figure 2. Through experiments, the bone scaffolds made of this material have good compatibility and mechanical properties.

Kawai et al. [65] printed FGS with PCL and β-TCP as the raw materials, which have controllable porosity, excellent degradability and mechanical strength. Animal experiments show that the degradation rate of the scaffold in vivo is higher than that in vitro, and the degradation rate of the proximal and distal segments is higher than that of the middle segment.

The degradation time of PLGA can be controlled by adjusting the monomer ratio, and its degradation products are the same as those of human metabolism. The 3D-printed PLGA scaffold by Xu et al. [66] can effectively solve the problem of craniomaxillofacial bone defects and is expected to cure patients with craniomaxillofacial bone defects. Zhang et al. [67] adopted the low-temperature deposition 3D printing technology to prepare PLGA scaffolds, which were proved to have excellent physical and chemical properties and nontoxic by experiments.

#### 2.2.2. Nondegradable Synthetic Polymer Materials

**Figure 3:**
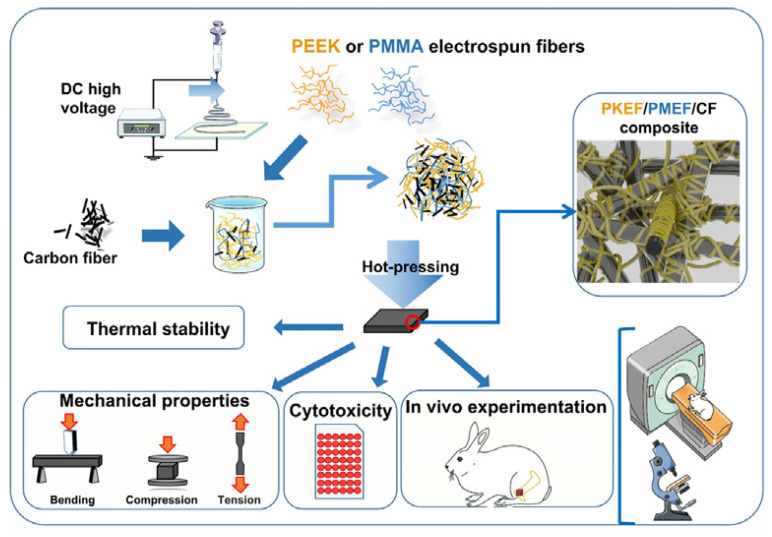
Preparation process and related tests of ternary composites [72].

Poly-ether-ether-ketone (PEEK), polyamide (PA) and other materials have good biocompatibility and an elastic modulus comparable to that of the human cortical bone, which can reduce the stress shielding after implantation in the human body. They are widely used as bone graft materials. The disadvantages of PEEK are that it is not biologically active and its surface osteogenic efficiency is low. The combination with other materials can solve these problems. Gu [68] synthesized PEK-CN through nucleophilic polycondensation, and blended nHA with PEK-CN by the solution method. Finally, PEK-CN, nHA/PEK-CN and porous nHA/PEK-CN scaffolds with good mechanical properties and good bone integration ability were prepared by melt deposition 3D printing technology. Li et al. [69] successfully fabricated porous SP-PEEK by FDM technology using PEEK as the raw material. SP-PEEK exhibited mechanical properties in the range of human trabecular bone and cortical bone, which can be simulated by adjusting the pore size and the number of pore layers. Human bone, which is beneficial for stress relief shielding, was tested and found that the SP-PEEK group with a pore size of 0.6 mm exhibited the best osteogenic performance in vitro. Polley et.al. [70] fabricated piezoelectric porous barium titanate (BaTiO_3_) and hydroxyapatite (HA) composite scaffolds by a 3D printing process. The printed scaffold demonstrated good cytocompatibility and cell attachment as well as bone-mimicking piezoelectric properties with a piezoelectric constant of 3 pC/N. This suggests that interconnected porous networks and micropores can be combined to create implant materials with improved bone regeneration potential that can enhance bone growth and vascularization. Zhao et al. [71] tested the biomechanical properties of 3D printed PEEK bone implants through tensile and bending experiments. The results showed that the implant had excellent mechanical properties, chemical properties and biocompatibility. Jia et al. [72] prepared PEEK/PMMA (polymethyl methacrylate)/CF (carbon fiber) ternary composites. The preparation process is shown in Figure 3. Mechanical tests show that the Young’s modulus of PEEK/PMMA/CF ternary composites is between 4.67 ± 0.38 and 6.37 ± 0.81 GPA, which is similar to human bones. Cytotoxicity tests in vitro showed that it was nontoxic. In vivo experiments show that the ternary composites show good histocompatibility without rejection or inflammation.

### 2.3. Natural Polymer Material

Materials such as sodium alginate, collagen, and chitosan (CTS) can be directly extracted from natural organisms, possessing good bio adhesion, biocompatibility, and excellent biodegradability [8,9,10]. Natural polymer materials have certain defects in mechanical properties, which can be improved by compounding with other materials [73,74,75].

Using gelatin (GEL) and stearyl acrylate (SA) as the raw materials, Yu et al. [76] prepared a GEL/SA gel scaffold using a three-dimensional biological printer, which had good biocompatibility and promoted the proliferation of human dental pulp cells and could be used as a dental regeneration scaffold. Yuan et al. [77] used silk fibroin (SF) and type II collagen (COL II) as the raw materials to prepare a composite scaffold with regular pore size and good permeability, which meets the relevant requirements of cartilage tissue engineering. Lu et al. [78] prepared naringin (NA-CTS)-CTS/HA composite and printed the composite scaffold in 3D. It can provide the necessary carrier for bone defect repair. NA can create a local osteogenic microenvironment and accelerate the growth of new bone tissue, with good bone repair performance. Luo [79] modified GEL/SA bio-ink by adding CNF. The test results showed that the modified bio-ink exhibited high bio-printing precision, flexible customization, and good biocompatibility in the bio-printing of cell components of the ligament–bone interface structure. Liu [80] prepared the copper-containing mesoporous bioactive glass composite scaffold. The results of cell experiments showed that the scaffold significantly promoted the proliferation of MC3T3-E1 cells, and it was a bone tissue engineering scaffold with great application potential. Sing et al. [81] Ti/Col I and Ti-Ta/Col I as the raw materials for the biphasic scaffold, which can improve the differentiation and proliferation of inoculated cells by mimicking the rigid bone phase and porous cartilage phase. The collagen can still obviously infiltrate into the scaffold, effectively enhancing the biological response, thus demonstrating the feasibility of the biphasic scaffold for bone tissue repair.

### 2.4. Future Prospective and Conclusions

All bone graft materials have at least one of the following biological characteristics: (1) Bone conductivity: providing a scaffold for the growth of blood vessels and the formation of new bones, such as tricalcium phosphate, hydroxyapatite and artificial polymer materials. (2) Osteo-inductive: containing osteogenic-inducing protein, which can stimulate the mesenchymal stem cells around the bone graft area to differentiate into chondroblasts or osteoblasts, and form new bone. Commonly used synthetic polymer materials such as polyglycolic acid and polylactic acid have certain osteo-inductive ability; Ceramics rich in calcium and phosphorus salts, chemically and heat-treated metals such as titanium and tantalum, etc. PLA/HA composite, etc. (3) Osteogenesis: containing osteoblasts (osteoblasts or bone progenitor cells), which can directly form new bones once implanted in a suitable environment. Autologous bone has all the above characteristics, and is recognized as an ideal bone implant. Improving the performance of bone instead of biomaterials to make it more similar to autogenous bone is still the key research direction [82]. Natural bone has complex composite structures and physiological characteristics, and the bone implant prepared by a single material cannot be matched. Through combing the current research, it is found that the composite use of different materials can reflect better mechanical properties and biological properties, and the composite of metal materials can improve the wear resistance of bone implants and reduce the toxicity of metal ions to cells in vivo [83]. The high polymer material composite can improve the toughness of the bone implant and reduce the brittleness and the like. The combination of bio ceramic and organic material can improve osteogenic capability. All these have indicated that the use of composite materials is an important direction for the future development of bone implants.

## 3. 3D Printing Technology

At present, the fabrication technologies of augmentation materials for bone tissue engineering scaffolds mainly include fusion deposition modeling (FDM), stereo lithography appearance (SLA), selective laser sintering (SLS), laser powder bed fusion (LPBF), and biological printing. Different molding methods have their own advantages and disadvantages, as shown in Table 2.

In addition to the five bone implant molding techniques described above, there are also direct ink writing (DIW), digital light processing (DLP), and selective laser melting (SLM). The current research results of these technologies are presented below.

### 3.1. Selective Laser Sintering

Selective laser sintering generally uses powder consumables, and through high-temperature laser sintering, the parts are formed by stacking layers in cooperation with the movement of the bottom plate. Ceng et al. [91] prepared a porous calcium phosphate scaffold with epoxy resin and biphasic calcium phosphate as the raw materials by SLS technology, and inoculated mouse embryonic cells on it for observation and CCK-8 (cell counting kit-8) detection. The results showed that the porous calcium phosphate scaffold had good biocompatibility. Ruben et al. [92] used SLS technology to prepare porous pure tantalum implants with interconnection. Compared with porous TC4, it has more excellent bone conductivity, higher fatigue strength and high ductility, which proves that porous tantalum implants with adjustable mechanical and biological properties can be produced by SLS. This is the first step towards a new generation of open porous tantalum implants manufactured by SLS. Guo et al. [93] used HA and PCL as the raw materials to prepare HA and PCL scaffolds by SLS technology, and then carried out mechanical experiments and MTT tests on them. It was found that HA/PCL scaffolds had low cytotoxicity and excellent mechanical properties, which could meet the requirements of implantation in vivo. Li et al. [45] prepared a bone scaffold suitable for the human body by SLS technology with Mg-based slurry as the raw material. It has high porosity and high strength and can be gradually degraded in vivo. It will be widely used in bone tissue engineering in the future.

**Figure 4:**
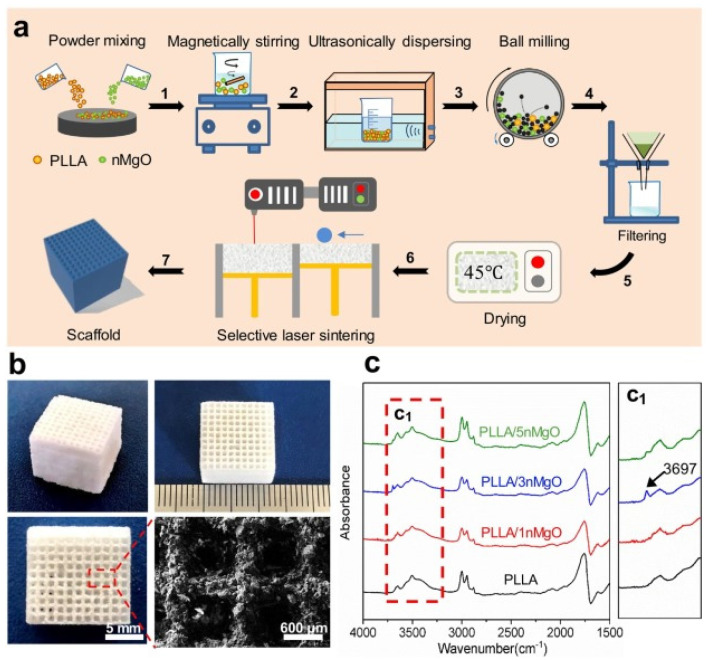
PLLA/n-MgO stent preparation and testing process. (**a**) The fabrication process of scaffolds, (**b**) the digital photographs of representative PLLA/nMgO scaffold, and (**c**) Fourier transform infrared spectrometer (FTIR) analysis results of the scaffolds [95].

Song et al. [94] prepared aliphatic polycarbonate/hydroxyapatite (a-PC/HA) composite scaffold by SLS technology. By adjusting the proportion of materials used, the best proportion of a-PC/HA composite material is 10 wt% HA. Experiments show that the scaffold has good bone conduction ability. A-PC/HA composite powder can be prepared by SLS technology for bone implantation. Jun et al. [95] prepared PLLA/nMgO scaffold by SLS molding technology with PLLA powder and NMGO as the raw materials. The manufacturing process is shown in Figure 4a, and the scaffold model is shown in Figure 4b, which presents a 3D porous structure with an average pore diameter of about 600 μm. The porous structure can provide an important microenvironment for cell adhesion, proliferation and nutrient exchange after implantation. Figure 4c is the infrared spectrum of PLLA and PLLA/n-MgO scaffold, which shows that some interactions have occurred between nMgO and PLLA, which promotes the mechanical strength of bone scaffold.

At present, the biggest problem of this technology requires a specific environment when printing, otherwise it may pollute the environment. The equipment is relatively large, and the price of equipment and materials is relatively high.

### 3.2. Fusion Deposition Modeling

**Figure 5:**
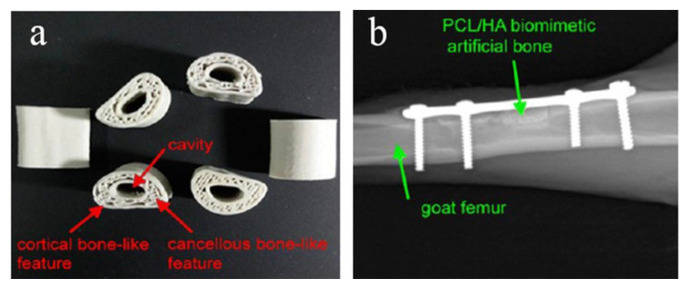
(**a**) Structure diagram of PCL/HA stent, (**b**) CT image of implantation in vivo [101].

Melting deposition molding uses high temperatures to melt the material into a liquid, and then uses a print head to extrude and solidify it, and finally forms a three-dimensional entity in a three-dimensional space. Dong et al. [96] printed custom Mg/PCL composite scaffolds with enhanced osteogenesis and biomineralization by FDM technology. Magnesium microparticles embedded in PCL-based scaffolds played an active role in enhancing biocompatibility, biomineralization, and biodegradability. When combined with 3 wt% Mg, the PCL-based scaffold exhibited the best bone repair ability in vitro and in vivo. In vitro experiments demonstrated that the Mg/PCL scaffolds had improved mechanical properties, good biocompatibility, and enhanced osteogenic and angiogenic activities. This suggests that 3D printed cell-free Mg/PCL scaffolds are a promising strategy for bone healing applications. Bulina et al. [97] found the technology of selective laser melting of hydroxyapatite has a great potential for 3D printing of medical biodegradable implants with complex shape and porous gradient structure at sufficiently fast printing speeds. He et al. [98] used polyvinyl alcohol (PVA) hydrogel and sheep vertebra bi-phase ceramics as the raw materials, and prepared the bone scaffold by FDM molding process, and tested its mechanical properties and biological experiments, respectively. The results showed that the bone scaffold had excellent mechanical properties, good biocompatibility and no toxicity. Zhang et al. [99] used PLA-HA composite material as the raw material and FDM printing technology to prepare circular scaffolds. The diameter and height of the scaffolds are 5 mm and 6mm, respectively. The aperture is 500 μm and the porosity is 60%. In the above study, the bone stromal cells were found to grow well on the scaffold without obvious infection. Jiao et al. [100] prepared the bone scaffold with HA/PCL as the raw material by FDM printing technology. The scaffold has a uniformly distributed porous structure and excellent mechanical properties. Xu et al. [101] used CT-guided FDM to fabricate the PCL/HA and PCL 3D artificial bones to mimic the natural goat femurs, as shown in Figure 5a. In order to study the performance of artificial bone in vivo, it was transplanted to the defect of goat femur, as shown in Figure 5b. The results show that it has good biocompatibility and biodegradability.

Senem et al. [102] used PCL as the raw material, prepared bone scaffold by FDM printing technology, and modified it employing nano hydroxyapatite (Hap) and polypropylene fumarate (PPE). Experiments show that the scaffold has a clear pore size, high mechanical strength, high porosity, controllable surface hydrophilicity (using PPF) and bone conductivity (using HAp) and excellent biocompatibility.

The surface accuracy of FDM molded parts is low, and the supporting structure needs to be designed and manufactured. Secondly, the molding time of this technology is too long, which cannot be achieved for emergency use.

### 3.3. Stereo Lithography Appearance

Stereophotography appearance is to focus the laser with specific wavelength and intensity on the surface of photocurable material, and make it be shaped point by point and face by face. Qin et al. [103] prepared ceramic slurry with 40% solid content based on acrylic acid, and successfully printed out tooth crowns and bridges by SLA technology. Through experimental measurement, it was found that its mechanical properties were better than human teeth. Ding et al. [104] used SLA technology to prepare a porous titanium alloy scaffold, implanted mouse cells into the alloy scaffold and observed the proliferation and differentiation of cells and found that the titanium alloy scaffold showed excellent biocompatibility and bone conductivity. Chen et al. [105] used light curing water-borne polyurethane as the raw material to prepare cartilage scaffolds by SLA technology. Experiments showed that these cartilage scaffolds can induce cartilage differentiation and are the best choice to replace damaged cartilage. Elomaa et al. [106] used PCL as the raw material to prepare bone tissue scaffold by SLA printing technology. The average porosity of the scaffold was 70.5 ± 0.8% and the average pore diameter was 465 μm. Pore networks are highly interconnected. Experiments show that PCL resin is very suitable for manufacturing solvent-free tissue engineering scaffolds by stereolithography. Zhang et al. [107] used 45S5 bioactive glass and mixed photosensitive resin as the raw materials to prepare 3D mesh scaffold by SLA printing technology. The scaffold has a rough surface with many holes with a diameter of 5 μm, which provides a place for cell growth and proliferation, and secondly serves as a channel for nutrient transport. The experimental results show that the scaffold has good biocompatibility. Tesavibul et al. [108] prepared the 45S5 Bio-glass scaffold by stereolithography, which has a honeycomb structure and excellent mechanical properties. The 3D printing technology based on lithography provides an excellent alternative to the existing manufacturing methods of bone implants and scaffolds, which can accurately control the 3D shape and pore structure. Senatov et al. [109] successfully printed porous scaffolds with PLA/15 wt% HA as the raw materials, and the average pore size and porosity of the scaffolds were 700 μm and 30 vol%. Experiments show that PLA/15 wt% HA porous scaffold obtained by 3D printing has a shape recovery rate of 98% and can be used as a self-adaptive implant for small bone defect replacement of SME.

The biggest challenge of this technology is that the software system is too complicated to operate, and it is difficult to get started, and its applicable file format is unfamiliar to most designers. Secondly, the products produced by this technology need to be sintered.

### 3.4. Laser Powder Bed Fusion

FPBF irradiates the surface of powdered raw materials with laser beams, and through layer-by-layer scanning, melting, solidification and final molding, the raw materials are instantly melted, cooled and solidified. Guo et al. [110] adopted LPBF technology to prepare bone tissue engineering scaffolds using HA and PCL as the raw materials. The bone scaffold prepared by the laser sintering technology has an interconnected 3D pore structure, and the porosity is as high as 80%, which can be observed by a scanning electron microscope. Jin et al. [111] prepared a bone scaffold by LPBF technology with mixed powder of nHA/polycaprolactam (PA6) as the raw material. The inner diameter of the bone scaffold is 800 μm, the outer diameter is 6mm and the height is 15 mm. In vivo experiments on the bone scaffold showed that the scaffold had higher cell adhesion and better cell proliferation, corresponding to a higher concentration of nHA, and no obvious cytotoxicity was observed. Yoo et al. [112] used LPBF technology to prepare bone tissue engineering scaffolds with complex structures using nHA as the raw material. The experimental results show that the compressive strength of the two-part structure scaffold is much higher than that of the normal human skeleton. The scaffold has the effects of promoting cell growth and inducing bone generation, and can promote the growth and healing of femoral defect tissues. Tan et al. [88] used LPBF technique to fabricate the lattice structure of TiNi and investigated the geometric integrity, microstructure evolution and phase transformation behavior of the lattice. Yin et al. [113] adopted the LPBF method to prepare porous titanium alloy scaffolds to improve the osteogenic effect of porous titanium. They also used GEL and nHA to construct scaffolds in titanium alloy pores. The LPBF technique enables titanium alloys to be independently designed as scaffolds with high porosity. The porosity of the macro-porous scaffold may exceed 80%. This not only provides enough space and sufficient nutrients for bone growth, but also weakens the stress shielding effect in the process of bone growth, thus better meeting the clinical requirements.

In the process of LPBF printing, when the high-energy laser beam hits the powder bed, local laser heating will cause the surface to boil and form a strong steam jet, which will make the melt surface sag, which is an unpredictable problem of LPBF process and a great challenge for the development of this technology.

### 3.5. Bioprinting

**Figure 6:**
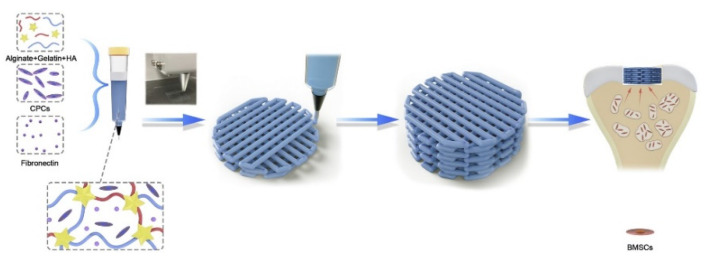
3D printing schematic diagram of chondrogenic progenitor cells (CPC) and fibronectin (FN) encapsulated in biofilm for repairing cartilage defects [117].

The most significant advantage of 3D bio-printing is that in the printing based on living cells, biological materials and growth factors, the living cells are added on the surface of the bone scaffold relative to other augmentation manufacturing technologies, so that more personalized macro and micro structures can be manufactured. Biological printing can be divided into ink-jet, extrusion, and laser-assisted [89]. Poldervaart et al. [114] prepared a bone tissue scaffold containing living cells and growth factors using 3D biological printing technology and added bone morphogenetic protein 2(BMP-2) to the bone scaffold. The release time of BMP-2 was controlled by hydrogel. The in vivo experiments showed that the bone scaffold prepared by this technology could realize the long-term release of BMP-2 through hydrogel, thus controlling the bone formation rate. Zhou et al. [85] used expanded chondrocytes in combination with PGA, PLA, and PCL to prepare artificial ears using 3D bioprinting technology. The results showed that the stent accurately reproduced the symmetrical 3D structure of the ear and had good mechanical properties. Ahn et al. [115] used PPF as the raw material and prepared a 3D porous bone scaffold using laser-assisted bio-printing, which realized the accurate construction of 3D microstructures by controlling the scanning speed. Kang et al. [116] used adipose-derived stem cells (ADSCs) and PCL/β-TCP as the raw materials and prepared a skull implant using a multi-nozzle 3D biological printing technology. Animal experiments show that the bone implant has excellent mechanical properties and osteogenic properties. Yang et al. [117] used 3D bioprinting technology to add cartilage-forming progenitor cells (CPC) and fibronectin (FN) to the ALG/Gel/HA composite hydrogel, as shown in Figure 6. The hydrogel prepared an active biofilm with uniform pores and effectively repaired cartilage defects by using a modified autologous matrix-induced cartilage formation (AMIC) technology.

Guo et al. [118] created a 3D printing scaffold functionalized with aggrecan to improve cell adhesion and maintain cell function on the surface of the scaffold. Compared with a common scaffold, the scaffold improves the quality of regenerated cartilage tissue during micro-fractures.

### 3.6. Other Printing Technologies

Sa et al. [119] used β-TCP and photosensitive acrylate resin as the raw materials to prepare the controllable porous β-TCP scaffold by DLP technology, with the preparation process shown in the figure. The scaffold has a compressive strength of up to 9.8 MPa and a porosity of 40%, has strong biocompatibility, and promotes cell adhesion and angiogenesis during bone regeneration. Yong et al. [120] used HA as the raw material and prepared the porous HA scaffold using DLP technology. The high-porosity 3D printed titanium stent constructed by Lim et al. [121] using the SLM method has good biocompatibility and bone conductivity in vivo. During the 6-week observation period, the proportion of new bone in the 3D-printed titanium stent was approximately 25%. However, radiologically and histologically, no differences in bone formation based on the three pore designs or implantation phase were observed.

### 3.7. Discussions before Future Prospective and Conclusion

Through sorting out the existing achievements of 3D printing technology applied to bone implants, it was found that the printing methods could be mainly divided into two categories. Some were based on laser technology, such as SLA and SLS. The laser was used to locally melt the material powder or other materials, and the three-dimensional parts were formed through layer-by-layer stacking. Post-processing the three-dimensional part becomes an implantable graft. This kind of printing method based on laser technology can print parts with complicated shapes. The surface quality of the parts is good and generally has high mechanical properties. However, the equipment which uses laser technology is generally large in size, high in energy consumption, requires a special environment and is high in cost. The other is that the material is melted by heating in the spray head, such as FDM, etc. The spray head moves along the cross-sectional profile and filling track of the part, and extrudes the melted material at the same time, so that the material can be solidified rapidly and bonded with the surrounding material, which is formed by stacking layer by layer. This technology does not need a laser, and the equipment volume is relatively small, simple to use and maintain, and low in cost. It does not need a special independent environment when printing and is relatively clean. However, the technology of the printing product’s surface is relatively rough, the forming speed is slow, and for larger products, it needs to increase the support when printing. Moreover, that spray head is easy to block and is inconvenient to maintain. Bio-printing technology is developed based on the above two technologies. There are three printing methods: inkjet printing, extrusion printing and laser assisted printing. Biological ink is used as the raw material to print. The printed product structure is more refined and the biological performance is more superior. In general, the bone implants printed by the current printing technology are inactive, and the biological printing technology is an important direction for breaking through the defect.

## 4. Optimization of Structure and Performance of Bone Implant

**Figure 7:**
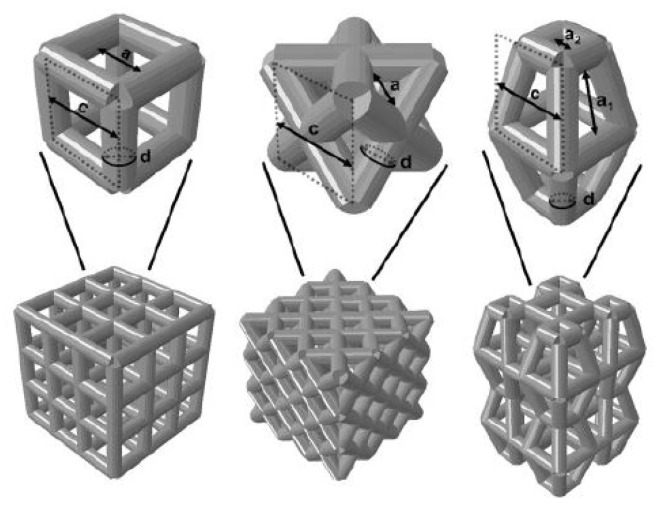
Upper Row: Parameterized models of the cubic structure (**left**), diagonally orientated struts (**middle**) and modified truncated pyramid (**right**) with the strut diameter (d), size of the basic cell (c) and pore size (a). Bottom Row: Mechanically tested scaffolds, consisting of 3 × 3 × 3 basic cells (cubic and diagonal design) and 2 × 2 × 2 basic cells for the modified truncated pyramid design (with additional connection elements). Struts are shown as cylindrical beams with their analytical cross-section [122].

The surface layer of natural bone was dense, with a porosity of about 4%, and the interior consisted of cancellous bone with a porosity of 50% to 90%. Since the pore structure inside the natural bone is very complex, and the bones in different parts even have differences in pore shape, pore radius, and porosity in different parts of the same bone, it is very difficult to construct a bone unit structure that is consistent with heaven and man bones artificially, but the bone unit structure is a key feature of bone implants, because the structure affects mechanical properties and biological reaction, as well as the flow of nutrients in the bone implants. The nutritional supply of bone implants is an important factor determining cell survival and reproduction. Therefore, the structural optimization of the bone unit is a key step in the development of bone implants. The research results in this field in recent years are introduced below. Wie et al. [122] parametrically designed the pore micro-unit of cortical bone by numerical optimization method. Figure 7 shows the optimized pore micro-unit of cortical bone. The experimental results show that numerical optimization can effectively reduce the amount of materials without affecting the biomechanical properties, thus reducing the quality of implants.

**Figure 8:**
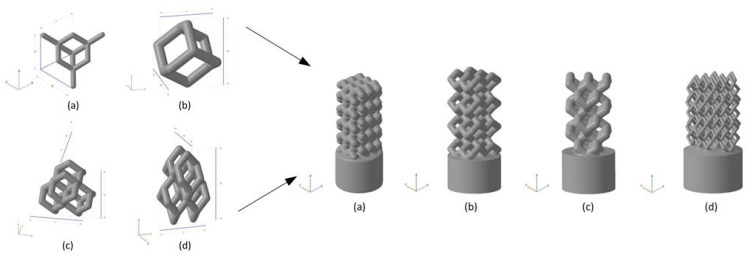
Unit cells used for implant modeling and the models of implants. Left: (**a**) UC001—the unit cell built on the basis of tetrahedral diamond structure (rib thickness = 0.4 mm, cell length (L) = 2.3 mm, cell width (W) = 2.3 mm, cell height (H) = 1.7 mm); (**b**) UC00202—the cell built on the basis of the UC001 unit cell by removing open ribs (rib thickness = 0.7 mm, L = 1.6 mm, W = 1.6 mm, H = 2.3 mm); (**c**) UC003—the cell built by replicating the UC002 unit cell along the X and Y axes by a factor of 2, with the offset along Z-axis by H/2, and along X-axis by W/2 (rib thickness of 0.7 mm, L = 3.5 mm, W = 3.5 mm, H = 3.8 mm); (**d**) UC004—the cell built by compressing the UC003 unit cell along X and Y axes by the factor of 2 (rib thickness =0.3 mm, L = 2.0 mm, W = 2.0 mm, H = 4.1 mm). Right: (**a**) BI001—the wireframe built by replicating the UC001 unit cell along three coordinate axes, by a factor of 3 along X and Y axes, and by a factor of 5 along Z-axis; (**b**) BI002—the UC002 unit cell is replicated by a factor of 2 along X and Y axes, and by the factor of 4 along Z axis; (**c**) BI003—the UC003 unit cell is replicated by a factor of 1 along X and Y axes, and by a factor of 3 along Z-axis; (**d**) BI004—the UC004 unit cell is replicated by a factor of 3 along X, Y, and Z axes [125].

Liu et al. [123] used HA as the raw material and prepared the HA bone scaffold using DLP method, with the model diagram shown in. The porosity of the scaffold was 49.8% and the pore size was between 300 and 500 μm. The in vitro biocompatibility of MC3T3 cells showed that the hydroxyapatite porous bone scaffold was nontoxic and could promote the adhesion, proliferation and differentiation of osteoblasts. Chen et al. [128] used light curing water borne polyurethane as the raw material and used stereo lithography apparatus technology to prepare the cartilage scaffold. It has been found in experiments that these cartilage scaffolds can induce cartilage differentiation and are the best choice for replacing damaged cartilage. Zhou [124] provided a unit cell model designed using TPMS (triply periodic minimal surfaces) curved surfaces and a bone scaffold model obtained through Boolean operations. It has good porosity and mechanical properties and is suitable for cellular attachment. Safonov et al. [125] prepared four kinds of macro-porous ceramic implants based on Al_2_O_3_ by using 3D printing technology. As shown in Figure 8, through experimental tests, the BI001 type implant has demonstrated effective compressive strength comparable to that of the trabecular bone, making it a promising bone substitute able to withstand high operating loads.

Pei et al. [52] combined 3D printing and microwave sintering to produce HA scaffolds with hierarchical macro and micropores. The authors tested the material in vivo and compared the results with a conventional porous HA made by H_2_O_2_ gas foaming method. New bone formation was observed for samples produced by 3Dprinting followed by microwave sintering, indicating that this methodology produces osteo inductive materials. On the other hand, the H_2_O_2_foaming samples did not show any bone formation, probably due to the lack of microporosity.

Ma et al. [126] prepared four groups of 3D bone scaffolds including PCL, PCL/PVAC (polymer vinyl acetate), PCL/HA and PCL/PVAC/HA for comparison through 3D printing technology. The experimental results including cell culture in vitro, material testing machine, and animal experiments on mechanical properties and biological characteristics showed that these 3D printing scaffolds had porous channel structure with a pore size of 375–475 μm and porosity of 74.1–76.1%. The PCL/PVAC/HA scaffold exhibited higher cell proliferation and bone formation rates than other groups. Liu et al. [127] combined the electrostatic spinning method with 3D printing technology to design and manufacture an HB stent. The scaffold was composed of upper and lower layers, with the upper layer serving as a barrier to prevent the invasion of surrounding fibrous connective tissue into the bone defect, and the lower layer designed for bone tissue regeneration. HB scaffolds can promote bone repair and regeneration in vivo, and form more new bone tissues in the rabbit bone defect model. Wang et al. [128] performed the optimal design of the porous medullary joint implant, and the results showed that the optimized porous medullary joint implant effectively prevented the fretting phenomenon of the implant under the biomechanical action after it was implanted into the body, and reduced the bone loss at the same time, showing the optimal porous skeletal joint implant designed by the author, with potential for clinical application.

Park et al. [129] prepared the pelvic implant using electron beam melting (EBM) printing technology and added horseshoe-shaped steel plates to fix the key parts. The results showed that the EBM printing technology could effectively reduce the loads on the bones and improve the mechanical strength of the pelvis.

**Figure 9:**
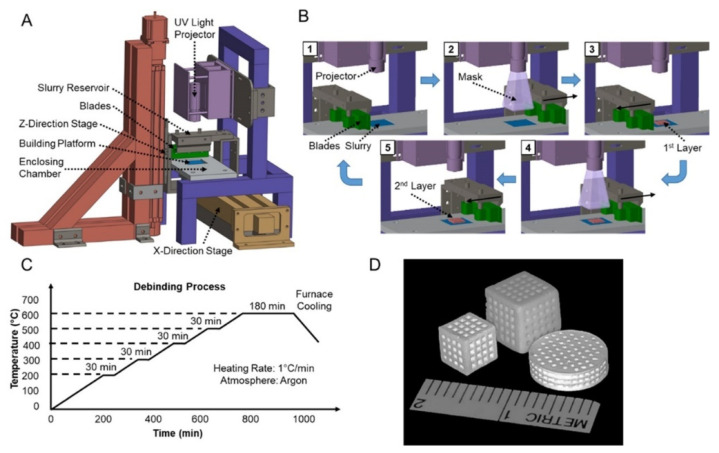
Diagram of β-TCP scaffold fabrication using SEPS. (**A**) 3D printer setup. (**B**) Layer-by-layer fabrication process. (**C**) Postprocess procedural de-binding protocol to remove excess binder resin solution from the β-TCP-printed component. (**D**) Photographs of 3D-printed β-TCP scaffolds in various shapes and sizes [135].

Bone implants prepared by 3D printing technology sometimes fail to meet the requirements, and the material of the bone implant can be surface modified by inorganic components or organic components or by changing the surface topography of the implant, thereby improving the osteogenic capability, bone conductivity, bacteriostasis, biocompatibility and the like of the bone implant to different degrees [130]. Weingartner et al. [131] printed a scaffold using PLC material and coated it with I Collagen. The biocompatibility experiment showed that PLC-GEL scaffold was suitable for cells. Cells adhere to the surface of the scaffold and grow apart. It has an acceptable strength of 68.49 ± 0.47 MPa as determined by the compressive strength. Zhao et al. [71] further improved the osteogenic property of the material by introducing amino groups on the surface of 3D-printed PEEK bone scaffold. Sun et al. [132] found the optimal filling angle and coating concentration through orthogonal experiments and prepared the personalized bone scaffold with multi-stage pore structure and porosity of 84% using β-TCP coated with I Collagen as the material and 3D printing technology. The mechanical and biological performance tests showed that the compressive property was similar to that of human cancellous bone, with good biocompatibility, and good osteogenic activity for BMSCs. Poh et al. [133] conducted a study on the performance of bioactive glass and strontium metal pairs for 3D printing scaffolds. 45S5 bioactive glass and strontium-substituted bioactive glass were, respectively, incorporated into PCL particles, and two cubic scaffolds, PCL/45S5 and PCL/Sr BG, were printed out by melt extrusion at 110 °C. Compared with the pure PCL scaffold, the composite scaffold has a high percentage of small-scale pore diameters, and the addition of the bioactive glass remarkably improves the compression elastic modulus of the scaffold, thereby being beneficial to application in hard tissue engineering. Zhang et al. [134] prepared MBG-β-TCP scaffold with graded pore structure and functional surface coating, which had high compressive strength and excellent apatite mineralization ability. MBG-β-TC scaffold can enhance the new bone-forming ability and the attachment of human umbilical vein endothelial cells in vivo, resulting in significantly enhanced expression of angiogenic genes. It indicated that the biological properties of the 3D printing scaffolds modified by MBG nano-layers could be effectively improved. Matthew et al. [135] used a hybrid method to combine 3D printed biodegradable and bone-conductive β-TCP with bone-inducible miR-200c to construct a synthetic bone graft, as shown in Figure 9. The 3D printed β-TCP scaffold was prepared by SEPS (styrene-ethylene/propylene-styrene block copolymer) process to produce a structure with reproducible microstructure, thus enhancing the bone conductivity of β-TCP. The 3D printed β-TCP scaffold is coated with collagen mixed with miR-200c, which can improve the transfection efficiency of miR-200c to rat and human bone marrow mesenchymal stem cells and increase the osteogenic differentiation of human BMSCs in vitro. In addition, miR-200c composite scaffold significantly enhanced the bone regeneration of critically size rat skull defects. These results strongly indicate that the bone graft combined with SEPS3D printed osteoconductive biomaterial scaffold and osteo-inductive miR-200c can be used as an excellent bone substitute for clinical treatment of large bone defects.

Nasrin et al. [136] studied the biological behaviors and bone supporting capacity of PCL, PCL/HA, PCL/BG and PCL/HA/BG four groups of 3D printing scaffolds. The experimental comparison showed that compared with PCL and the control alone, the bio-ceramic coated scaffolds exhibited a more suitable surface for cell adhesion and proliferation, as well as an effective potential for inducing bone conduction and bone integration. The PCL/HA/BG scaffolds showed higher cell viability and bone formation in vitro compared to the other groups.

In order to make the printed bone implant closer to the natural bone, the inside of the printed bone implant needs to be of a porous structure, so that the elastic modulus of the bone implant prepared by different materials can be adjusted to be suitable for a human body to avoid stress rejection, and cells in the body can be provided with a space for growth and reproduction to promote the generation of new bone. The mechanical properties and biological properties of the bone implant can be improved by modifying the surface of the bone implant or the printing material.

## 5. 4D Printing

Tibbits first put forward the concept of 4D printing in 2013, that is, adding the time dimension to 3D printing. The 4D printed bionic structure obtained by printing stimuli in response to biomaterials will change shape in response to external or internal stimuli. 4D bio-printing technology can manufacture complex organizational structures and dynamically control shape transformation as needed [137].

Kirillova et al. [138] provided an advanced 4D biological manufacturing method, which used polymer hydrogel as the raw material to manufacture hollow self-folding tubes, and controlled their diameter and structure with unprecedented high resolution. The mouse experiment shows that the 4D printing method will not have any negative influence on the vitality of printed cells, nor will it reduce the vitality of cells. Such a 4D printing method allows the dynamically reconfigurable architecture with adjustable functions and responsiveness to be produced by selecting appropriate materials and cells. Wu et al. [139] developed a self-repairing hydrogel that can be used for 4D printing and consists of biodegradable polyurethane (PU) nanoparticles and photo/thermal responsive gelatin-based biomaterials. The 4D bio-printed hydrogel cryopreserved (−20 °C or −80 °C) showed cell proliferation similar to that of the non-cryopreserved control after being awakened at 37 °C and recovered in shape. This kind of 4D bio-printable and self-repairing hydrogel with shape memory and low-temperature storage characteristics can be used for customized bio-manufacturing.

## 6. Future Prospective and Conclusions

Through summarizing the achievements of 3D printing technology in bone tissue engineering in recent years, we have found that it has great potential in this field. A qualified bone implant needs to meet the following tissue characteristics, cell structure pore size and porosity, mechanical properties, biocompatibility, osteo-conductivity and osteo-induction, and angiogenesis potential, which is still a challenge for the current research. Only by studying the relationship between these tissue characteristics and the manufacturing method, can we have the opportunity to develop more appropriate biomaterials that more closely approximate the hierarchical structure of natural bone. Through summarizing the printing materials of the bone implant, it is found that compared with a single material, the composite material has great advantages for preparing the bone implant, not only can the excellent properties of a single component material be preserved, but also the requirements that a single material cannot meet can be achieved through the complementary advantages of various materials. Of course, there are other ways to improve the properties of a single material, such as chemical modification and blending, and they also have good results. The composite material can be personalized according to the requirements of people; for example, the mechanical properties of the bone implant can be improved, the degradation rate of the bone scaffold in vivo can be changed, and the bone implant has good biological stability. At present, the printing method of bones mainly includes the printing method based on laser technology, the printing method using a nozzle to melt materials and extrude and superpose, and the biological printing technology using bioactive materials. Bioprinting is currently the most promising printing modality for preparing active implants. The bone implant can be applied to the human body by changing the internal structure and porosity to adjust the elastic model, mechanical properties, biological properties, etc., and the mechanical properties and biological properties of the bone implant can be improved by utilizing materials such as organic substances and inorganic substances to modify bone materials or the surface of the bone implant.

At present, there are still many problems in 3D printing technology. The internal structure of the printed bone implant is regular, while the internal structure of the natural bone is irregular, and the performance of different parts of the same bone is also different, which is incomparable to the artificial bone. Second, bionic bone implant designs with combined immunomodulatory properties should be considered to enhance natural bone regeneration and thus improve clinical outcomes. Now, the 3D printing product of the 3d printing product is characterized by one-time molding and hardly changes due to changes in the environment. Liquid material biological ink is a self-defined material, and relevant growth factors and nutrients can be added according to the needs of products to meet the needs of cell proliferation and differentiation. However, the environment in the human body is relatively complex, and many reaction mechanisms are still unclear. Although the existing biological ink can print out bone tissue, it cannot be used for human transplantation, and still needs more exploration and try. Based on this, 4D printing technology should be born. At first, 4D printing was considered as “3D printing and time”. With the deepening of research, the definition of 4D printing has been gradually improved. Currently, the generally accepted theory is that when the structure of 3D printing is subjected to a predetermined stimulus, its properties and functions will change over time. Common stimuli in the biomedical field are temperature, water and magnetic fields. Using biological printing materials that can be adjusted according to the external stimulation, 4D printing can produce the engineered tissue structure with “activity” and more complex structure, which is very similar to the natural tissue structure. In the future, 4D printing technology will be widely used in the medical field to solve the problems in the weaving process and drug delivery.

## Figures and Tables

**Table 1:** General properties of commonly used biomaterials in BTE [14,15,16,17,18,19,20,21,22,23,24,25,26].

Material Type	Material	Advantages and Disadvantages	Ref.
Inorganic Materials-Metals	Titanium and its alloys	High strength, bioinert, low density, not biodegradable, low modulus of elasticity	[14]
	Magnesium and its alloys	High strength, low density, good rigidity, good degradability, poor corrosion resistance	[15]
Inorganic biomaterials Bio-ceramics	Hydroxyapatite (HAp)	Biocompatible osteoconductive/osteo-inductive, brittle, low mechanical strength, slow resorption rate	[16]
	β-tricalcium phosphate (β-TCP)	Biocompatible, highly resorbable, osteoconductive/osteo-inductive brittle	[17]
	Bioactive glasses	Bioactive, high strength and toughness, elastic modulus, wear resistance, fast degradation rates which can be overcome by incorporating different ions in the glass structure	[18]
Natural polymers	Gelatin	Biocompatible, non-immunogenic, biodegradable, liquefies at physiological temperatures, poor mechanical properties	[19]
	Collagen	Non-cytotoxicity, low antigenicity response, crosslinking capacity, enzymatic biodegradability, complex structure	[20]
	Silk fibroin (SF)	Biocompatible, elastic, excellent mechanical strength, slow degradability	[21]
	Hyaluronic acid (HA)	Biodegradable, biocompatible, viscoelasticHighly hydrophilic, not mechanically stable, slow gelation rate	[22]
	Chitosan	Biodegradable, good antithrombogenic and hemostatic action, muco-adhesion, analgesic effect, antifungal activity, insoluble in water	[23]
	Poly (lactic-co-glycolic acid) (PLGA)	Biocompatible, biodegradable	[24]
	Polycaprolactone (PCL)	Biocompatible, biodegradable, excellent mechanical properties	[25]
Inorganic–organic composite biomaterials	SF/β-TCP; SF/HAp	Mechanical properties enhancement, high cell attachment and proliferation, increased in vivo response and new bone formation	[26]

**Table 2:** Introduction of widely used bone implant molding methods based on 3D printing technology [84,85,86,87,88,89,90].

Forming Method	Materials Used	Advantages and Disadvantages	Ref.
SLS	Synthetic polymers, polymer-ceramic/inorganic composites (e.g., PCL/TCP, PLLA/Mg, PCL/HA)	A broad variety of biomaterials, no need for assistance and post-processing; Thermal distortion that can cause shrinking and warping issues	[84,85]
SLA	Limited materials: epoxy/HA, poly (trim-ethylene carbonate)/nHA, poly (ethylene glycol-co-depsipeptide) hydrogel	High accuracy, complex 3D structure, cell inclusion; Limited to photosensitive resin; layers cause stair-stepping instead of smooth surface	[86]
FDM	Synthetic polymers (e.g., PCL, PLA, PLGA)	High porosity, complete pore interconnectivity, control over porosity, and pore size; print quality is not as good as SLA or SLS; limited to thermoplastic polymers; problems with warping and minor shrinking	[87]
LPBF	Inorganic nonmetals, metal powders and organic polymer materials	Fast processing speed and high efficiency; Due to the high temperature generated during the molding process, the organic polymer material may be degraded, and there will be residual raw materials.	[88]
Bioprinting	Bio-ink (Natural biomaterials such as ALG, GEL, HA and ODM)	Biocompatible and biodegradable; the molding speed is slow, the mechanical properties are poor, and subsequent improvement is required.	[89,90]
